# Application of C-Terminal Clostridium Perfringens Enterotoxin in Treatment of Brain Metastasis from Breast Cancer

**DOI:** 10.3390/cancers14174309

**Published:** 2022-09-02

**Authors:** Amita R. Banga, Peace Odiase, Kartik Rachakonda, Amar P. Garg, Samuel E. Adunyah, Girish Rachakonda

**Affiliations:** 1 Department of Biotechnology, School of Biological Engineering & Sciences, Shobhit Institute of Engineering & Technology, Meerut 250110, India; 2 Department of Microbiology, Immunology and Physiology, Meharry Medical College, Nashville, TN 37208, USA; 3 Undergraduate Studies, School of Arts and Sciences, University of South Florida, Tampa, FL 33620, USA; 4 Department of Biochemistry, Cancer Biology, Neuroscience & Pharmacology, Meharry Medical College, Nashville, TN 37208, USA

**Keywords:** Claudin-4, clostridium perfringens enterotoxin (CPE), cancer, metastasis, brain cancer

## Abstract

**Simple Summary:**

Brain metastasis occurs in primary cancers, such as breast cancer, and is correlated with mortality. There are limited options available for treatment, but *Clostridium perfringens* Enterotoxin (CPE) and its interaction with Claudin-4, a possible diagnostic biomarker for breast cancer, can provide a molecular pathway basis for the development of treatment options for metastatic brain cancer. Analysis of the literature reveals that Claudin-4 plays an important role as a receptor for CPE, allowing for the disruption of cell membrane permeability, an influx of calcium ions, and subsequent cell death. The negligible presence of Claudin-4 in normal brain cancer cells and the high abundance of Claudin-4 in breast cancer cells metastasized to the brain, allow for the targeted binding of CPE to tumor cells in the brain. We show that the C-terminal of CPE conjugated to nanoparticles that cross the blood–brain barrier could serve as a drug delivery tool to treat metastatic cells in the brain.

**Abstract:**

Claudin-4 is part of the Claudin family of transmembrane tight junction (TJ) proteins found in almost all tissues and, together with adherens junctions and desmosomes, forms epithelial and endothelial junctional complexes. Although the distribution of Claudin-4 occurs in many cell types, the level of expression is cell-specific. Claudin proteins regulate cell proliferation and differentiation by binding cell-signaling ligands, and its expression is upregulated in several cancers. As a result, alterations in Claudin expression patterns or distribution are vital in the pathology of cancer. Profiling the genetic expression of Claudin-4 showed that Claudin-4 is also a receptor for the clostridium perfringens enterotoxin (CPE) and that Claudin-4 has a high sequence similarity with CPE’s high-affinity receptor. CPE is cytolytic due to its ability to form pores in cellular membranes, and CPE treatment in breast cancer cells have shown promising results due to the high expression of Claudin-4. The C-terminal fragment of CPE (c-CPE) provides a less toxic alternative for drug delivery into breast cancer cells, particularly metastatic tumors in the brain, especially as Claudin-4 expression in the central nervous system (CNS) is low. Therefore, c-CPE provides a unique avenue for the treatment of breast–brain metastatic tumors.

## 1. Introduction

Clostridium perfringens (*C. perfringens*) is a spore-forming gram-positive bacterium that naturally exists in the environment in soil, sewage, and the guts of humans and animals [1]. *C. perfringens* is known for its role as a common disease-causing agent of food poisoning, diarrhea, and other gastrointestinal illnesses [2]. *Clostridium perfringens* type-A bacteria produce several toxins, prominent among these is *Clostridium perfringens* enterotoxin (CPE) which is the causative agent in antibiotic-associated diarrhea and symptoms linked to common gastrointestinal food-borne illnesses [3]. CPE creates pores in intestinal and colonic epithelial cells, rendering them permeable to an influx of Ca^2+^ and consequent cellular damage [4]. 

Functionally, CPE consists of two main domains: the pore-forming NH2-terminal region and the Claudin-binding c-CPE. By forming pores in the cytoplasmic membrane of mammalian host cells, the N-terminal region of CPE disrupts osmotic equilibrium and mediates a cytotoxic effect [5]. CPE and Claudin-4 have aromatic and hydrophobic interactions rather than hydrogen bonding, and Tyr306 is crucial to this binding [6]. CPE binds initially to the receptor proteins, creating a small (90 kDa) precursor complex which forms a larger SDS-resistant complex (155 kDa) that induces altered permeability in the cell membranes of sensitive mammalian cells [7]. The resulting modifications in cellular membrane permeability generate a calcium influx, triggering rapid apoptosis or oncosis. A hexameric prepore on the cell membrane is assembled first and then beta-hairpin loops generate a beta-barrel that infixes into the membrane, intensifying calcium influx through the active pore. Subsequent cell death causes fluid and electrolyte loss and damage to the intestines [8].

## 2. Molecular Targets of CPE in the Human Body

**Figure 1:**
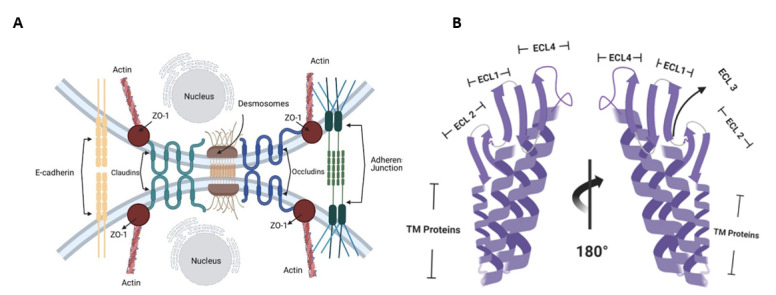
(**A**) Claudin receptors are a major component of tight junctions, preventing the entrance of molecules into the cells and maintaining cellular permeability and osmotic potential. (**B**) Structural domains of Claudin-4.

Claudins are important components of tight junctions (TJs), which help in maintaining the integrity of cell membranes and preserving the polarity across cellular sheets (Figure 1A).

TJs establish a fence that hinders lateral diffusion of membrane proteins and lipids, thereby sustaining the differential composition of basolateral and apical regions, providing a means through which regulatory signals can be traduced to and from cells via the actin cytoskeleton. Claudins localize to endothelial and epithelial cell sheets along with occludin, zonal occludens -1 (ZO-1), and junctional adhesion molecules (JAMs) [9]. Proteins in the Claudin family are small (20–27 kDa) transmembrane proteins found in many organisms, ranging from nematodes to humans, and share a significant identity [10]. They have four transmembrane regions, two of which are extracellular loops (Figure 1B) [11]. The tubular arrangement of tumor epithelial cells is associated with Claudins because Claudin deletion mutants lead to tumors with decreased tubular arrangement [12]. Claudins thus alter cellular proliferation [12,13]. Due to the important roles Claudins play in hindering the metastasis and proliferation of cancer cells, Claudins are plausible targets for therapeutic intervention.

**Figure 2:**
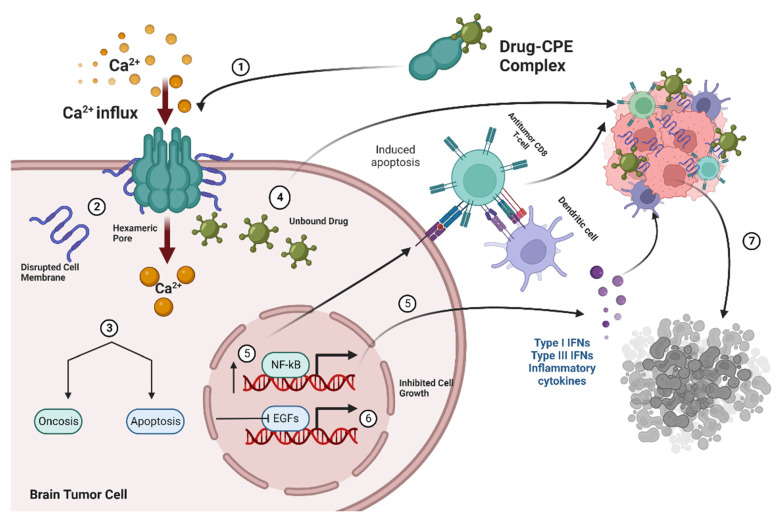
(**1**) Drug–CPE complex interacts with Claudin-4 receptors in brain metastatic tumor cells and forms a hexameric pore causing increases in influx of Ca^2+^, osmotic dysregulation, and membrane disruption (**2**), which leads to apoptosis or oncosis (**3**,**4**). Unbound drugs are delivered into tumor cells and further mediate cell destruction upon binding by exerting antitumor effects on cancer cells while (**5**,**6**) the modulation of growth factor signaling and nfKB increase cell death by decreasing cell proliferation and increasing expression of inflammatory cytokines and immune cells (**7**).

Claudin-4 TJ proteins are high-affinity receptors for CPE that create pores in intestinal and colonic epithelial cells, rendering them permeable to an influx of Ca^2+^ and consequent cellular damage (Figure 2) [5].

The implications of these interactions have led CPE to be considered as a suitable and effective therapeutic option for cancer. CPE structurally comprises three domains: C-terminal domain I, which binds to receptors, domain II, which oligomerizes and inserts into the cytoplasmic membrane, and domain III, which modifies CPE morphology after binding and membrane insertion [14]. The CPE polypeptide binds via its COOH-terminal region (c-CPE) to the second extracellular loop (ECL-2) in Claudin-4, its high-affinity receptor, and other Claudins as well [15].

## 3. Application of CPE and Claudin-4 Interactions in Treatment of Brain Metastasis from Breast Cancer

### 3.1. Brain Metastasis Treatment Options

Brain metastasis (BM) is the most commonly occurring form of intracranial tumors and is a typical complication of other primary malignancies such as melanoma, breast, and lung cancer [16]. BM is associated with cancer mortalities: approximately 10% to 26% of deaths in cancer patients are correlated with the development of BM [17]. There are approximately 98,000 to 170,000 new annual diagnoses of brain metastases in the United States, and few of these cases can be cured using currently available therapies [18]. Current therapies include the use of corticosteroids, whole-brain radiation therapy (WBRT), surgical procedures with or without WBRT, radiotherapy to the surgical bed, and stereotactic radiosurgery (SRS) [19]. The survival rates of WBRT are dismal (2–4 months) and are typically used in recursive partitioning analysis (RPA III) for patients with serious complications, including but not limited to encephalopathy and cerebral edema [18]. The use of corticosteroids also presents complications, such as myopathy, immune suppression, mood alterations, and severe psychiatric disturbances [18,19]. Surgical resection in RPA I/RPA II patients who had single lesions and minimal or controlled systemic disease has proved successful, with less than 1% of surgeries resulting in complications. However, only about 15% of patients are eligible as BM may not be localized to a particular region of the brain [18]. SRS targets single/multiple lesions with high beam radiations and can be used to treat deep-seated tumors. SRS is contraindicated in excessively large target lesions or too many lesions for practical use, and seizures have been reported as a complication in 40% of BM treatments [18,20]. Considering these facts, more options are needed for BM treatment. 

Drug delivery is a crucial aspect of drug development. Many drugs are incapable of crossing the cell membrane or passing from one cell to another. A drug’s ability to traverse epithelial and/or endothelial cell membranes through either transcellular or paracellular routes is vital to its pharmacokinetics and biodistribution, especially across the blood–brain barrier [21]. Transcellular drug delivery occurs via simple diffusion or by active transport via a receptor or transporter on cytoplasmic membranes [22]. The existence of variability in gene expression profiles of transporters among tissues introduces difficulty in delivering drugs to a target tissue using a specific transporter. The paracellular delivery of drugs occurs by disrupting the TJ barrier by loosening the tight junctions’ scaffold [23]. This process naturally occurs in the intestines to absorb more nutrients after a meal. Absorption enhancers that dilate TJs, letting drugs permeate the intercellular spaces of epithelial cell membranes, such as chelators and surfactants, can be useful for the delivery of various molecules [24]. However, they have low tissue specificity and provoke severe side effects, including desquamating the intestinal epithelium which permanently alters its barrier capacities, and thus they have limited use [24]. Claudin-4 is an important tight junction functional protein, normally localized in the cell membrane where, through binding, it regulates the paracellular transport of solutes. Claudin-4 expression is typical in epithelial tumors and is elevated in epithelial malignancies and metastasis [24].

### 3.2. Claudin-4 Expression Patterns

**Figure 3:**
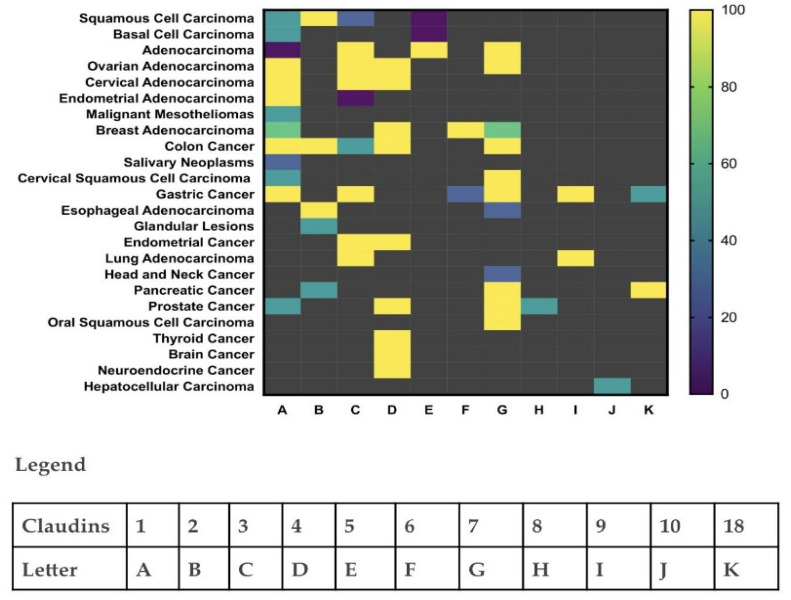
Reconstructed heatmap with graph prism showing Claudin protein expressions as biomarkers for cellular differentiation and indicators for phenotypic expression of the epithelia from several research studies [26,27,28,29,30,31,32,33,34,35,36,37,38,39,40,41]. Claudin protein expression patterns in varying cancer types can therefore provide information for cancer identification.

Claudin expression levels vary with tumor types, tumor aggression, and invasion potential [13] (Figure 3). Deletion mutations of Claudins are crucial in the progression and invasive potential of tumors as Claudins regulate the permeability of the epithelial membrane to the flux of ions and small molecules in order to maintain epithelial polarity and homeostasis [12,25]. Claudins are also highly involved in cell–cell signaling via actin linkage, cellular differentiation, cell proliferation, and the transcription of genes [10]. As Claudin expression levels increase in cells, these highlighted processes also increase, including cell migration, drug resistance, and cell-cycle progression, dependent on the presence of anchorage [26]. Hence, Claudin dysfunction contributes significantly to the impairment of cell adhesion, remodeling of the cytoskeleton, morphogenesis, detachment of tumor cells, and distant metastasis. Moreover, reduced border regulation and increased paracellular permeability increase the progression, proliferation, viability, migration, and invasion of tumor cells by allowing the passage of necessary growth factors and nutrients. Claudin expression patterns can also indicate tumor type as the distinct fashion in which the Claudin proteins aggregate in cells depends on the cell type and location [27,28,29,30,31,32,33,34,35,36,37,38,39,40,41,42,43,44] (Figure 3). Claudin-4 is a marker of cellular differentiation and an indicator for phenotypic expression of the epithelia and thus a plausible target for therapeutic intervention for several cancers and their precursors.

In colon epithelia, Ca^2+^ levels, electrophysiological reports of Ca^2+^ uptake, and calcium-detection microscopy have confirmed that CPE interaction with Claudin-4 in TJs determines the extent to which CPE can disrupt TJs and mediate cytotoxicity [45]. Through pore formation in plasma membranes upon binding to Claudin-4 and the subsequent influx of Ca^2+^, CPE causes fluid and electrolyte loss, apoptosis, and oncosis via the activation of calmodulin and calpain [46]. In breast cancer, CPE treatment has also shown Claudin-4 dependence [47]. The tumorigenesis, progression, and pathology of breast cancer have been linked to different expressions of Claudins, and Claudin-4 has proved very powerful in predicting survival [28,29,30]. An evaluation of lymph node metastases has shown that Claudin-4 overexpression is a predictor of poorer outcomes in breast cancers [28]. The brain has little or no Claudin-4 expression in contrast to brain tumors where Claudin-4 is overexpressed. Only cells of epithelial origin express Claudin-4, whereas brain cells are of mesenchymal origin. Therefore, it is mainly metastatic brain tumors that express Claudin-4. This helps to successfully target a brain tumor and not normal CNS tissue [48].

## 4. Use of C-Terminal CPE as a Therapeutic Agent in Brain Metastasis from Breast Cancer

### 4.1. Claudin-4 and C-CPE Interactions in Cancer Cells

CPE toxin is lethal for cells high in Claudin-4 expression. CPE gene transfer in vitro and in vivo, using the bacterial wild-type CPE cDNA (wtCPE) or optCPE cDNA, selectively killed tumor cells overexpressing Claudin-4. The expression of optCPE was more efficient and demonstrated quick cytotoxic activity, heightened by the bystander effect of CPE release. Up to 100% cytotoxicity in tumor lines expressing Claudin-4 specifically was reported 72 h following the transfer of CPE genes [49]. In vitro CPE treatment of breast cancer cell lines (MDA-MB-468, MCF-7, NT2.5-Luc) and normal human astrocytes led to a dose-dependent and accelerated cytolysis solely in breast cancer cells. The extent of cytolysis was limited by Claudin-4 expression [48]. Furthermore, the intracranial administration of CPE into breast metastatic cancer cells in the brain—using the murine model with brain tumors from the human breast cancer cell line MDA-MB-468 and the murine breast cancer cell line NT2.5-Luc. —improved survival significantly in comparison to mice treated with PBS [48]. Hence, CPE in its native form can be advantageous in treating various cancers with overexpressed Claudin CPE receptors, notwithstanding that immunogenicity and bystander toxicity pose a problem. Moreover, CPE impairment of tight junctions has now been shown to result in stemness, epithelial–mesenchymal transition (EMT), activation of the Yes-associated protein (YAP), a transcriptional coactivator in oral squamous cell carcinoma, and intracellular displacement of Claudin-4 to the cytoplasmic membrane [49]. These results imply that CPE might heighten the malignant transformation of cells via YAP activation.

**Figure 4:**
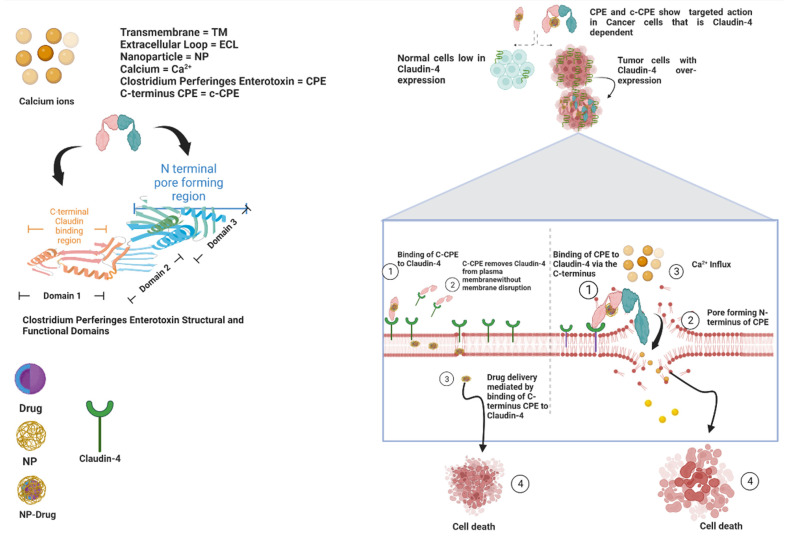
Diagrammatic representation of the mechanisms of CPE and c-CPE interaction with Claudin-4 and structural and functional domains of both CPE and Claudin-4.

Alternatively, the Claudin-binding C-terminal domain of CPE (c-CPE) can be employed to reversibly regulate TJs and enhance permeability in order to permit the delivery of solutes and chemotherapeutic agents across epithelial cell sheets [50]. In the absence of the toxic pore-forming N-terminal of CPE, c-CPE protein complexes are effective and a better candidate than native CPE for cancer therapy. C-CPE peptide comprises amino acids 184 to 319 and contains the receptor-binding region at amino acids 290 to 319. The superimposition of a homology model of the human Claudin-4 apo form on the Claudin-4•c-CPE fusion protein structure revealed substantial changes in conformation when c-CPE bound with Claudin-4 in both the ECL1 and ECL2 domains [51]. c-CPE binds to and removes Claudin-4 from TJs without redistributing the unbound Claudins and damaging the plasma membrane, allowing for drug entry and absorption to occur (Figure 4) [52].

This mechanism is much slower and more reversible because it is dependent on the c-CPE concentration [34]. Consequently, c-CPE can act as a Claudin modulator to open TJs for improved delivery of drugs across tissue membranes by disrupting TJs to increase paracellular permeability without destroying the plasma membrane integrity and incurring cytotoxicity [53,54]. For example, Polysialic acid nanoparticles conjugated to CPE peptides (C-SNPs) were recently developed for targeted therapy against pancreatic cancer. C-SNPs with loadings of doxorubicin (DOX-C-SNPs) collocate and target Claudin-4 in pancreatic cancer cells, disrupting TJs while being significantly reduced in normal pancreatic cells [55]. Similarly, nanoparticles made of the biocompatible and biodegradable polymer, Poly(lactic-co-glycolic-acid) (PLGA-NPs), altered with c-CPE (c-CPE-NPs) and transferred intraperitoneally, significantly inhibited tumor growth in ovarian cancer cells without much activity in normal ovarian cells [56]. Moreover, c-CPE mutants have shown exclusive binding capabilities to Claudin-4. For instance, c-CPE 194 is a c-CPE mutant that binds only to Claudin-4 and enhances the effectiveness of anticancer agents. In well-differentiated HPAC (a pancreatic cancer cell line) duct epithelial cells, c-CPE 194 interrupted barrier functions without changing Claudin-4 expression while increasing MAPK phosphorylation. c-CPE 194 also augmented the cytotoxicity of gemcitabine and S-1 (two anticancer drugs), reduced the expression of Claudin-4, and improved MAPK activity in a poorly differentiated pancreatic cancer cell line, PANC-1. In normal human pancreatic duct epithelial cells, c-CPE 194 diminished Claudin-4 expressions and improved the MAPK activity without impacting the cytotoxicity of the anticancer drugs [57]. In breast cancer cells, c-CPE fused to protein synthesis inhibitory factor (PSIF) showed cytotoxicity leading to dose-dependent cell death in MCF-7 human breast cancer cells but not in mouse fibroblast L cells due to the presence of Claudin-4 in the breast cancer cells and the absence in mouse fibroblast L cells [58]. c-CPE also shows Claudin-4 dependence in mediating its cytotoxicity in breast cancer cells [58]. c-CPE binding, which correlated with the expression of Claudin-4, aids the cytotoxic actions of carboplatin and paclitaxel by sensitizing epithelial ovarian cancer cells. c-CPE significantly increased tumor suppression through the inhibition of tumor cell proliferation and acceleration of tumor apoptosis when added to carboplatin and paclitaxel compared to when the anticancer agents were used independently [54]. Modulation of Claudins by c-CPE increases drug absorption by 400-fold when compared to sodium caprate, an absorption enhancer utilized in the clinic [59]. Additionally, Claudin-4 positive human ovarian carcinoma cells experienced a 6.7-fold elevation in toxicity when treated with a fusion of recombinant c-CPE and tumor necrosis factor (TNF) than with TNF alone [60]. Recombinant c-CPE-proteins can thus be developed as TJ modulators for enhanced delivery of drugs into breast cancer and brain metastatic cells overexpressing Claudin-4. The treatment of breast cancer cells and brain metastasis with c-CPE in its various forms is effective because Claudin-4 is steadily expressed in those cancers. Furthermore, Claudin-4 expression in normal areas of the central nervous system (CNS) is negligible, which restricts apoptosis exclusively to brain tumor cells and hinders tumor growth [48]. c-CPE thus has theranostic value.

### 4.2. Crossing the Blood–Brain Barrier

The blood–brain barrier (BBB) hinders the passage of neurotoxic substances and drugs into the brain. c-CPE has been modified in studies to transiently open and cross the BBB by binding to Claudin-5 (Cldn-5) in the endothelial TJs that form the paracellular barrier in BBB [61,62,63]. Recently, several different approaches have been undertaken to enhance the uptake of chemotherapeutic agents into malignant tumors, including conjugating drugs to micelles, liposomes, dendrimers, and nanoparticles—which may be proteinaceous, polymeric, or inorganic—that can cross the BBB [64]. The uniqueness of each drug delivery system lies within its bioavailability, hydrophilicity, biocompatibility, membrane permeability, and biodegradability [65]. Notably, nontoxicity and the capacity for localization to tumor sites are important when considering the suitability of drug delivery techniques in cancer treatment. Preferably, an ideal drug delivery vehicle would be nontoxic, able to solubilize in the body, permeable to the tissue barriers, biodegradable, and capable of providing direct, targeted, and sustained effects against tumor cells [66]. Using Claudin-4 as a target for fluorescent molecules, c-CPE bound to NP (c-CPE NP) can be further localized and administered intraperitoneally (IP) in breast cancer cells to significantly lessen systemic toxicity than with the same dose administered intravenously. Further, CPE-expressing vectors can be utilized to transfer genes intratumorally for selective suicide gene therapy of chemotherapy-resistant cells in tumors expressing Claudin-4. For instance, in ovarian cancer resistant to IP chemotherapy, fluorescent FITC conjugated to CPE accumulated predominantly in the ovarian tumor and not the normal ovarian cells [39]. Furthermore, c-CPE-NPs were used to deliver *Diphtheria Toxin Subunit-A* (DT-A), a therapeutic agent, via the p16 promoter highly expressed in ovarian tumors. The p16 DT-A vector enclosed in CPE-NPs (p16 DT-A c-CPE-NPs) led to cell death in ovarian cancer in vitro. IP injections of p16 DT-A c-CPE-NPs inhibited tumor growth significantly more than using control NPs in mice harboring chemotherapy-resistant tumors (*p* = 0.041) [56]. Nanoparticles conjugated with c-CPE thus depict a nontoxic binal-targeting approach for treating chemotherapy-resistant cancer cells via selective gene therapy and for drug delivery to non-chemo-resistant brain metastatic cancer cells. To date, there is not extensive research that shows the interaction between c-CPE conjugated to drugs in breast cancer metastasis in the brain. However, several drugs have been shown to concentrate in breast cancer metastasis in the brain, crossing the impaired BBB caused by tumorigenesis [67]. Such drugs include trastuzumab, Zr trastuzumab, C-paclitaxel, C-doxorubicin, and C-lapatinib [68,69,70,71]. As stated, c-CPE-NPs conjugated to drugs like doxorubicin and paclitaxel have shown efficacy in other cancer cell types, such as ovarian and pancreatic cell lines. Thus, c-CPE mutants that bind to Cldn-5, a major TJ protein in the BBB, and are conjugated with drug-NPs can potentially serve as a novel technique to direct cancer therapy to breast cancer metastasis in the brain.

## 5. Concluding Remarks

Claudins are important proteins in embryonic development and contribute to normal cellular physiology, playing significant roles in maintaining intestinal homeostasis, cell signaling and proliferation, tumorigenesis, and tumor inhibition. The dysregulation of Claudin transmembrane proteins is hence associated with many cancers of epithelial origin as the loosening of cellular adhesion compromises the structural integrity and functional efficacy of TJ complexes in cells of both the endothelium and epithelium during tumorigenesis. These observations suggest that these proteins are potentially useful as biomarkers for cancer diagnosis and can serve as targets for therapy. Various Claudin levels are changed in the development of tumors and most of the Claudin levels are elevated. Of the 27 Claudins, Claudin-4 shows a significant change in tumors and is overexpressed in primary breast cancers. Elevated levels of Claudin-4 were seen in invasive and metastatic breast tumor types. Claudin-4 is a high-affinity receptor for CPE and strongly binds the COOH-terminal of the CPE protein. Once bound, Claudin-4 changes the permeability of the membrane and causes cellular apoptosis. As a result, CPE has the potential for treating various tumors, such as breast tumors, and CPE therapy is suitable for diverse brain metastases without causing toxicity in the CNS. Since many tumor cells show elevated levels of Claudin-4 and Claudin-4 has a significant binding efficiency to CPE, CPE is applicable for drug delivery. The toxicity of CPE, however, makes c-CPE a better candidate for cancer therapy and is useful for improving reversible and concentration-dependent drug absorption due to its lessened toxicity and its fewer antigenic determinants. c-CPE conjugated to nanoparticles and delivered intraperitoneally is by far the most successful method shown to localize c-CPE to tumor cells highly expressing Claudin-4 and to further reduce cytotoxicity. To further aid in crossing the blood–brain barrier (BBB), c-CPE mutants that transiently bind to Claudin-5, the most predominant tight junction protein in the paracellular seal, can aid drug delivery. Increased knowledge of the Claudin-4•c-CPE structure and the conformation changes induced from c-CPE binding to Claudin-4 can also provide insights to help design relevant c-CPE mutants as Claudin modulators. Claudin-4•c-CPE interactions can also aid the creation of technologies that modify TJs containing Claudins for improved metastatic brain cancer treatment from primary cancers such as breast cancers that overexpress Claudin-4.
